# Increased Production of γ-Aminobutyric Acid from Brewer’s Spent Grain Through *Bacillus* Fermentation

**DOI:** 10.4014/jmb.2210.10051

**Published:** 2022-12-26

**Authors:** Tao Kim, Sojeong Heo, Hong-Eun Na, Gawon Lee, Jong-Hoon Lee, Ji-Yeon Kim, Do-Won Jeong

**Affiliations:** 1 Department of Food and Nutrition, Dongduk Women’s University, Seoul 02748, Republic of Korea; 2 Department of Food Science and Biotechnology, Kyonggi University, Suwon 16227, Republic of Korea; 3 Department of Food Science and Technology, Seoul National University of Science and Technology, Seoul 01811, Republic of Korea

**Keywords:** Brewer’s spent grain, *Bacillus velezensis* DMB06, *Bacillus licheniformis* 0DA23-1, fermentation, γ-amino butyric acid

## Abstract

Brewer’s spent grain (BSG) is a waste product of the beer industry, and γ-aminobutyric acid (GABA) is a physiologically active substance important for brain and neuron physiology. In this study, we used the bacterial strains *Bacillus velezensis* DMB06 and *B. licheniformis* 0DA23-1, respectively, to ferment BSG and produce GABA. The GABA biosynthesis pathways were identified through genomic analysis of the genomes of both strains. We then inoculated the strains into BSG to determine changes in pH, acidity, reducing sugar content, amino-type nitrogen content, and GABA production, which was approximately doubled in BSG inoculated with *Bacillus* compared to that in uninoculated BSG; however, no significant difference was observed in GABA production between the two bacterial strains. These results provide the experimental basis for expanding the use of BSG by demonstrating the potential gain in increasing GABA production from a waste resource.

## Introduction

Brewer’s spent grain (BSG) is a byproduct filtered from beer and other fermented liquids made from barley [1]. As beer consumption increases, the amount of available BSG also increases. The main ingredients of BSG are carbohydrates and proteins, which have not been fermented into alcohol during the brewing process. BSG also contains organic acids, vitamins, enzymes for alcohol production, and yeast [1, 2]. Most BSG is generally discarded as a waste product, although some are used as animal feed and fertilizer. However, recent studies have highlighted the physiologically active ingredients in BSG that regulate blood sugar levels, while attention has also been drawn to its angiotensin-I-converting enzyme inhibitory activity, and anti-inflammatory and antiallergenic properties [3, 4]. Overall, preliminary studies on the health benefits of BSG have focused on improved functionality by adding BSG or improved functional components through the fermentation of BSG. However, detailed studies on bioactive compounds that can be further isolated and produced from BSG are still lacking [5, 6].

γ-Aminobutyric acid (GABA) is a free amino acid with four carbons and is widely distributed in microorganisms, plants, and animals [7-9]. GABA is biosynthesized by decarboxylation of L-glutamic acid by the catalytic action of glutamate decarboxylase [10], or via putrescine from arginine by the proteins encoded by the *puuABCD* operon [11]. GABA is an inhibitory neurotransmitter of the central nervous system present in the brain and spinal cord of mammals, and it is known to improve brain blood flow, increase oxygen supply, and promote brain metabolic functions, thereby stabilizing nerves [12-14]. In addition, various other physiological activities, such as diuretic and antidiabetic effects, relief of anxiety and depression, and improvement of memory and learning ability, have been reported for GABA [14-16]. Therefore, studies are being conducted on the health benefits of foods that contain GABA, such as fermented milk, green tea, cheese, and vinegar [17]. Efforts to improve fermenting microorganism strains and develop novel fermentation processes are actively underway to increase the value of using GABA in functional foods and medicines. In addition, efforts are being made to strengthen functionality and increase the product value of fermented foods, such as soybean products, shrimp, cheese, makgeolli (Korean rice wine), and kimchi, by using microorganisms that produce GABA in the starter cultures for these products [18-22].

In our previous study, the biosynthesis genes for GABA production were identified in four *Bacillus* spp. by comparative genomic analysis, and these species were applied to soybean fermentation to determine the GABA production [9]. *B. licheniformis* and *B. velezensis* increased the production of GABA and glutamic acid during soybean fermentation. In our other previous studies, *B. velezensis* strain DMB06 and *B. licheniformis* strain 0DA23-1 were separated from fermented soybeans and selected as potential starter candidates through safety and functional evaluation [23, 24]. Here, the presence of GABA-producing genes was confirmed through genomic analyses of these two selected starter candidate strains, and their GABA production was assessed during the fermentation of BSG.

## Materials and Methods

### BSG, Bacterial Strains, and Culture Conditions

BSG was provided internally by Seoul National University of Science and Technology. In brief, brewing was done at a lab-scale with solid malt [a combination of Weyermann Pale Ale Malt (Germany) and Briess Dark Chocolate Malt 420L (USA)]. The brew was centrifuged (1,000 ×*g*, 4°C, 15 min), and the remnants were freeze-dried. *B. velezensis* DMB06 and *B. licheniformis* 0DA23-1 were used as starter strains for BSG fermentation [23, 24]. The strains were cultured in Tryptic Soy Agar (BD, USA) and Tryptic Soy Broth (TSB; BD) for 18 h at 37°C for maintenance.

### Genomic Analysis and Metabolic Pathway Prediction

Two strains, *B. velezensis* DMB06 and *B. licheniformis* 0DA23-1, previously selected as starter candidates, were used for genomic analysis [23, 24]. Genomic information on *B. velezensis* DMB06 (GenBank accession no. CP083763) and *B. licheniformis* 0DA23-1 (GenBank accession no. CP031126) was registered in the National Center for Biotechnology Information database (http://ncbi.nlm.nih.gov/genomes). Metabolic pathways were predicted using Rapid Annotations using the Subsystems Technology (RAST) server for SEED-based automated annotation [25]. The predicted metabolic pathways of the strains were confirmed using the iPath (ver. 3) module [26], and CLgenomics ver. 1.55 software.

### BSG Fermentation Using *Bacillus*

BSG was suspended in distilled water at a concentration of 10% (w/v) and cooled to room temperature after autoclaving at 121°C for 15 min. *Bacillus* for fermentation was precultured in TSB, and approximately 10^6^ CFUs/ml were inoculated into the suspension of BSG, followed by incubation at 50°C for 2 days.

### Determination of pH, Acidity, Reducing Sugar, and Amino-Type Nitrogen Concentration

Ten milliliters of the BSG fermentation sample were diluted with 90 ml of sterilized water and filtered through sterilized cheesecloth. The filtrates were analyzed for pH and acidity. The pH was measured using a pH meter (MP220, USA). The acidity was measured using phenolphthalein as an indicator with 0.01 M NaOH according to the General Test Method (6.12.2.3) of the Food Code of the Ministry of Food and Drug Safety, Republic of Korea. Reducing sugar was determined by the 3,5-dinitrosalicylic acid method using glucose as a standard substance after diluting the BSG fermentation [27].

Amino-type nitrogen content was analyzed by the formaldehyde titration method [28]. Ninety-five milliliters of distilled water was added to 5ml of the BSG fermentation sample, followed by homogenization and centrifugation at 10,000 ×*g* for 5 min. Distilled water (10 ml) and neutral formalin (10 ml) were added to the supernatant (10 ml) adjusted to pH 8.4 by adding 0.1 M NaOH. The final titrated volume was used to calculate the amino-type nitrogen content. Distilled water was used as the test blank. All experiments were conducted in triplicate.

### Viable Bacteria Monitoring in BSG

Ten milliliters of the BSG fermentation sample was completely mixed with 40 ml of deionized water for 5 min, filtered through Whatman filter papers (No. 2; GE Healthcare Life Sciences, USA), diluted 10-fold continuously with saline, spread on an agar plate, incubated at 37°C, and viable bacteria were then measured. The number of colonies was enumerated on Plate Count Agar (BD), and all experiments were repeated three times.

### Analysis of GABA from BSG

Supernatant from the BSG fermentation (100 μl) was obtained by centrifugation (8,000 ×*g*, 5 min, 4°C), followed by dansylation at 80°C for 40 min after addition of 1 M sodium carbonate–sodium bicarbonate buffer (pH 10.0; 200 μl), dansyl chloride dissolved in acetone at 20 mg/ml (100 μl), and distilled water (600 μl). Then, 10%(v/v) acetic acid (100 μl) was added to terminate the reaction. After centrifugation, the reaction solution was filtered with a 0.2-μm syringe filter (Merck Millipore, USA) and injected into a high-performance liquid chromatography (HPLC) system for analysis at 254 nm and 30°C [29]. A JASCO HPLC system (JASCO LC-2000 Plus Series, Japan) with a photodiode array detector, an autosampler (JASCO AS-2055 Plus), and an AccQ-Tag C18 column (3.9 × 150 mm, 4 μm; Waters Corporation, UK) was used. Eluent A was prepared by mixing tetrahydrofuran, methanol, and 50 mM sodium acetate–acetic acid buffer (pH 6.2) at a ratio of 1:15:84 (v:v:v), and eluent B was methanol. Eluents A and B were applied at a flow-rate of 1 ml/min for 5 min, 16 min, and 4 min at ratios of 80:20, 45:55, and 0:100, respectively [29]. The GABA content was calculated using calibration curves determined at five concentrations with GABA standard material (Sigma-Aldrich, USA). All experiments were conducted three times.

### Statistical Analysis

Duncan’s multiple range test followed by one-way ANOVA were used to evaluate significant differences between the average values obtained from the GABA analyses. *p* < 0.05 was considered statistically significant. Maximum variation rotation was applied to visualize the differences between the physicochemical components of BSG inoculated with bacteria. All statistical analysis was performed using the SPSS software package (version 27.0; SPSS, IBM, USA).

## Results

### Genomic Analysis of Potential GABA Production by *Bacillus* Strains

**Fig. 1:**
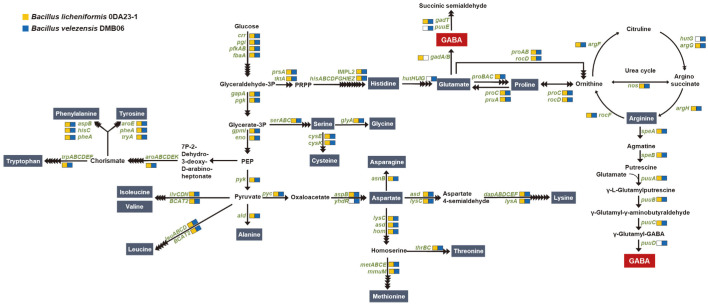
Predicted biosynthesis pathways of amino acids and γ-aminobutyric acid (GABA) in *Bacillus velezensis* DMB06 and *B. licheniformis* 0DA23-1. In the pathways, the names of enzyme-encoding genes and amino acids are depicted in green and blue boxes, respectively. The black arrows correspond to potential reactions catalyzed by enzymes encoded in the genomes of the *Bacillus* strains.

*Bacillus velezensis* DMB06 and *B. licheniformis* 0DA23-1 originated from fermented soybeans and were selected as starter candidates because of their enzymatic activities (*e.g.*, protease activity), following safety evaluation [23, 24]. To explore the potential of using these strains for GABA production from BSG, we first undertook analysis of their genomes. Complete genome sequences of *B. velezensis* DMB06 and *B. licheniformis* 0DA23-1 were used for genomic analysis of potential production of GABA and amino acids. The genome of *B. velezensis* DMB06 contained the genes for synthesis of 20 amino acids, while B licheniformis 0DA23-1 contained genes for the synthesis of 16 amino acids, from glucose (Fig. 1, Table S1). Strictly speaking, strain 0DA23-1 did not possess the *hutHIUG* operon, which is responsible for conversion of histidine to glutamate (Fig. 1); therefore, B licheniformis 0DA23-1 cannot produce arginine, glutamate, glutamine, or proline from glucose, because glutamate is a precursor of glutamine, arginine, and proline. However, genes for the biosynthesis of arginine, glutamine, and arginine from glutamate were retained in the strain, so these amino acids could be produced if glutamate were provided to the cell. The gene possession results for the amino acid biosynthetic pathway are consistent with our previous study [9]. Therefore, it was assumed that the biosynthetic genes and production of amino acids are not strain-specific, but species-specific.

There are two pathways to produce GABA, from the precursors glutamate and putrescine, respectively. In the former, GABA is produced from glutamic acid by decarboxylation catalyzed by glutamate decarboxylase [30]. In the latter, GABA is produced through putrescine in arginine [11]. The genome of *B. licheniformis* 0DA23-1 contains the genes encoding glutamate decarboxylase, *gadA/B* (BLDA23_RS06375). *B. velezensis* DMB06 has the genes to produce GABA from putrescine, notably γ-glutamyl-γ-aminobutyrate hydrolase, encoded by puuD (LAZ97_RS03000), which may convert γ-glutamyl-GABA to GABA (Fig. 1). These results for the GABA synthesis pathways are consistent with previous genomic analyses of *Bacillus* spp. [9].

In summary, the genomes of *B. licheniformis* 0DA23-1 and *B. velezensis* DMB06 contain genes required for the synthesis of GABA from glutamate and putrescine, respectively. Therefore, we applied these strains as starter cultures for GABA production from BSG.

### Physicochemical Constituent Analysis of BSG

Changes in pH and acidity were measured in BSG inoculated with *B. velezensis* DMB06 or *B. licheniformis* 0DA23-1 as a starter strain at 50°C for 2 days at intervals of 24 h. In preliminary experiments in which BSG was fermented by *Bacillus*, 50°C was the temperature that resulted in the highest GABA production, hence that temperature was used in our experiments.

**Fig. 2:**
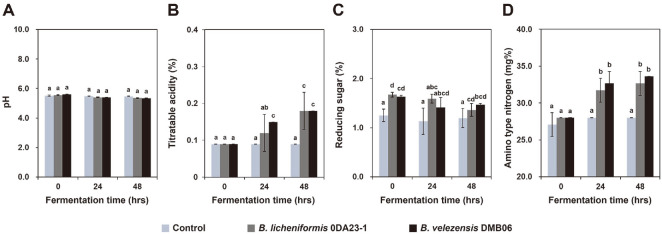
Changes in pH (A), acidity (B), reducing sugar (C), and amino-type nitrogen content (D) during fermentation of brewer’s spent grain (BSG) by *B. velezensis* DMB06 or *B. licheniformis* 0DA23-1. Different letter indicates significant difference at *p* < 0.05 using Duncan’s multiple range test.

The pH of the inoculated fermentation decreased during the experiment, but there was no dramatic change (Fig. 2A), nor was there a significant difference between the two strains. After 24 h of fermentation, the acidity had increased significantly (Fig. 2B), but after that, it increased only slightly (no further significant change).

In the BSG samples inoculated with *Bacillus*, the content of reducing sugar was higher than that in the control group, and the content of reducing sugar decreased during the fermentation (Fig. 2C) because the reducing sugar was used by the added microorganisms as a source of carbohydrate. Amino-type nitrogen increased as the fermentation progressed in the cultures inoculated with *Bacillus* (Fig. 2D), indicating an increase in free amino acids derived from BSG because of the enzymatic activity of the added microorganisms.

**Fig. 3:**
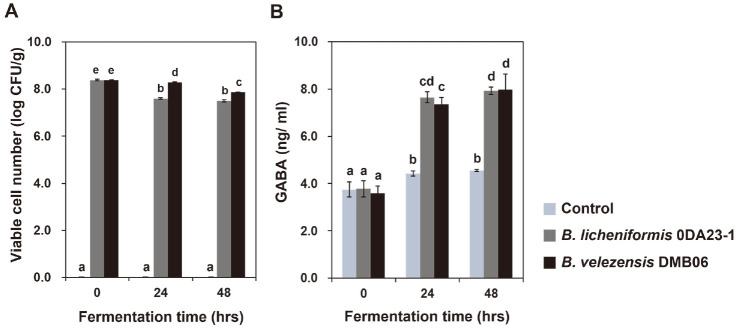
Viable *Bacillus* number (A) and GABA (B) production in BSG by *B. velezensis* DMB06 or *B. licheniformis* 0DA23-1. Different letter indicates significant difference at *p* < 0.05 using Duncan’s multiple range test.

The number of viable *Bacillus* decreased as the fermentation time increased (Fig. 3A). This was probably because the temperature used (50°C) was not optimal for the growth of *Bacillus* spp.

### Effect of *Bacillus* Species on GABA Production

We quantitatively determined GABA production from BSG by the two *Bacillus* species using HPLC. In BSG not inoculated with *Bacillus*, the GABA content was 8.35 ± 0.70 ng/ml at the start of the experiment, and 9.85 ± 0.24 ng/ml after 24 h. In BSG inoculated with *B. velezensis* DMB06, the GABA content after 24 h was 17.03 ± 0.51 ng/ml compared with 8.42 ± 0.76 ng/ml at the start of the experiment. In BSG inoculated with *B. licheniformis* 0DA23-1, the GABA content after 24 h was 15.66 ± 1.64 ng/ml compared with 7.97 ± 0.70 ng/ml at the start of the experiment (Fig. 3B). Through these experiments, we showed that *B. velezensis* DMB06 and *B. licheniformis* 0DA23-1 can enhance GABA production from BSG by fermentation.

## Discussion

**Fig. 4:**
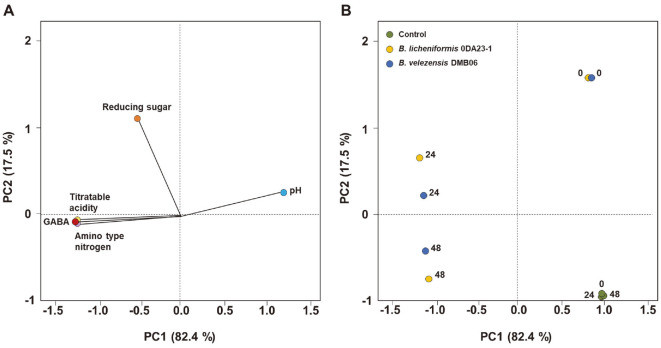
Principal component analysis loadings for fermentation of BSG by *B. velezensis* DMB06 and *B. licheniformis* 0DA23-1; (A) physicochemical factors and (B) factor scores. Numbers (0, 24, and 48) indicate the incubation times of samples in h.

To analyze the differences between *B. velezensis* DMB06 and *B. licheniformis* 0DA23-1 in GABA production during BSG fermentation, statistics on the pH, acidity, reducing sugar, amino-type nitrogen, and GABA content were subjected to PCA (Fig. 4). In a PCA factor loading plot, acidity, amino-type nitrogen, and GABA contents were located in the third quadrant of the PC2 dimension. After 48 h of fermentation, BSG inoculated with either strain separated from uninoculated BSG, but there was little difference between the strains. In conclusion, both strains had a significant effect on GABA generation from BSG.

We expected that *B. velezensis* DMB06 would be superior to *B. licheniformis* 0DA23-1 in GABA production, because *B. velezensis* DMB06 has the genes enabling production of glutamate from the glucose remaining in BSG (Fig. 1). *B. licheniformis* 0DA23-1 does not have the genes for production of glutamate from glucose, though it does have the genes needed for production of GABA from glutamate. However, there was in fact no significant difference in GABA production between the two strains. Therefore, we assume that GABA was generated from glutamate in the BSG (Fig. 3). Glutamate was detected in BSG by Jin *et al*. [31].

The world’s beer production has been approximately 2 billion hectoliters in recent years, producing byproducts such as waste grains, hops/trubs, and yeast. Waste grains account for approximately 85% of the by-products [32]. In Korea, the production of BSG is increasing, and the cost of disposing of it is also increasing, so we are looking for ways to use this material. The present study confirms the possibility of using BSG to produce GABA. Producing physiologically active substances using microorganisms from BSG, a type of waste, is considered necessary for sustainability.

## Supplemental Materials

Supplementary data for this paper are available on-line only at http://jmb.or.kr.
