# Supplementary material for: Increased Production of γ-Aminobutyric Acid from Brewer’s Spent Grain Through Bacillus Fermentation
Source: J Microbiol Biotechnol. 2022 Dec 26;33(4):527–32. doi: 10.4014/jmb.2210.10051 (PMC10164731; doi:10.4014/jmb.2210.10051)
Supplement: Supplementary file 1 [file jmb-33-4-527-supple.pdf]

https://doi.org/10.4014/jmb.2210.10051

Table S1. List of gene related with amino acids and GABA biosynthetic pathways.

Gene	E.C. No.	Product name	*B. licheniformis* 0DA23-1	*B. velezensis* DMB06
**GABA**				
speA	4.1.1.19	γ-Glutamylputrescine Synthase	BLDA23_RS09215	LAZ97_RS08225
speB	3.5.3.11	Agmatinase	BLDA23_RS21060	LAZ97_RS19045
puuA	6.3.1.2	Type I Glutamate--Ammonia Ligase	BLDA23_RS10795	LAZ97_RS09705
puuB	1.4.3.-	γ-Glutamylputrescine Oxidase	BLDA23_RS06920	LAZ97_RS05485
puuC	1.2.1.-	γ-Glutamyl-Aminobutylraldehyde Dehyderogenase	BLDA23_RS04115	LAZ97_RS03910
puuC	1.2.1.-	γ-Glutamyl-Aminobutylraldehyde Dehyderogenase	-	LAZ97_RS04115
puuC	1.2.1.-	γ-Glutamyl-Aminobutylraldehyde Dehyderogenase	BLDA23_RS21245	LAZ97_RS14730
puuC	1.2.1.-	γ-Glutamyl-Aminobutylraldehyde Dehyderogenase	BLDA23_RS12200	LAZ97_RS10670
puuD	3.5.1.94	γ-Glutamyl-γ-Aminobutyrate Hydrolase	-	LAZ97_RS03000
gadA/B	4.1.1.15	Glutamate Decarboxylase	BLDA23_RS06375	-
**Amino acid**				
yhdR	2.6.1.1	Aspartate Aminotransferase	-	LAZ97_RS05080
aroE	1.1.1.25	Shikimate Dehydrogenase	-	LAZ97_RS04140
hutG	3.5.3.8	Formimidoylglutamase	-	LAZ97_RS20030
hutH	4.3.1.3	Histidine Ammonia-Lyase	-	LAZ97_RS20015
hutI	3.5.2.7	Imidazolonepropionase	-	LAZ97_RS20025
hutU	4.2.1.49	Urocanate Hydratase	-	LAZ97_RS20020
pruA	1.2.1.88	L-Glutamate γ-Semialdehyde Dehydrogenase	-	LAZ97_RS19230
prsA	2.7.6.1	Ribose-Phosphate Pyrophosphokinase	BLDA23_RS00335	LAZ97_RS00320
prsA	2.7.6.1	Ribose-Phosphate Pyrophosphokinase	BLDA23_RS00450	LAZ97_RS00435

cysK	2.5.1.47	Cysteine Synthase	BLDA23_RS00455	LAZ97_RS00440
cysE	2.3.1.30	Serine O-Acetyltransferase	BLDA23_RS00635	LAZ97_RS00625
aroK	2.7.1.71	Shikimate Kinase	BLDA23_RS01885	LAZ97_RS01710
pruA	1.2.1.88	L-Glutamate γ-Semialdehyde Dehydrogenase	BLDA23_RS01900	LAZ97_RS01730
rocD	2.6.1.13	Ornithine--Oxo-Acid Transaminase	BLDA23_RS02125	LAZ97_RS20425
rocF	3.5.3.1	Arginase	BLDA23_RS02135	LAZ97_RS20415
lysC	2.7.2.4	Aspartate Kinase	BLDA23_RS02325	LAZ97_RS02020
serB	3.1.3.3	Phosphoserine Phosphatase	BLDA23_RS02805	LAZ97_RS02515
nos	1.14.14.47	Nitric-Oxide Synthase	BLDA23_RS04250	LAZ97_RS04025
aroD	4.2.1.10	3-Dehydroquinate Dehydratase I	BLDA23_RS05470	LAZ97_RS04145
prsA	2.7.6.1	Ribose-Phosphate Pyrophosphokinase	BLDA23_RS06695	LAZ97_RS05275
serC	2.6.1.52	3-Phosphoserine/Phosphohydroxythreonine Transaminase	BLDA23_RS06745	LAZ97_RS05325
asnB	6.3.5.4	Asparagine Synthase	BLDA23_RS07225	LAZ97_RS05670
mmuM	2.1.10	Homocysteine S-Methyltransferase	BLDA23_RS07320	LAZ97_RS05725
dapC	2.6.1.17	N-succinyldiaminopimelate aminotransferase	BLDA23_RS07405	LAZ97_RS05830
argF	2.1.3.3	Ornithine Carbamoyltransferase	BLDA23_RS07420	LAZ97_RS05845
metB	2.5.1.48	Cystathionine γ-Synthase	BLDA23_RS07820	LAZ97_RS06190
metC	4.4.1.13	Cysteine-S-Conjugate Beta-Lyase	BLDA23_RS07825	LAZ97_RS06195
proA	1.2.1.41	Glutamate-5-Semialdehyde Dehydrogenase	BLDA23_RS08440	LAZ97_RS07445
metE	2.1.1.14	Methyltetrahydropteroyltriglutamate--Homocysteine Methyltransferase	BLDA23_RS08490	LAZ97_RS07495
dapD	2.3.1.117	2,3,4,5-Tetrahydropyridine-2,6-Dicarboxylate N-Succinyltransferase	BLDA23_RS08975	LAZ97_RS07955
pyc	6.4.1.1	Pyruvate Carboxylase	BLDA23_RS09325	LAZ97_RS08335
IMPL2	3.1.3.15	Histidinol-Phosphatase	BLDA23_RS09735	LAZ97_RS08725
pfkA	2.7.1.11	6-Phosphofructokinase 2	BLDA23_RS09080	LAZ97_RS08045

asd	1.2.1.11	Aspartate-Semialdehyde Dehydrogenase	BLDA23_RS10305	LAZ97_RS09275
lysC	2.7.2.4	Aspartate Kinase	BLDA23_RS10310	LAZ97_RS09280
dapA	4.3.3.7	4-Hydroxy-Tetrahydrodipicolinate Synthase	BLDA23_RS10315	LAZ97_RS09285
tktA	2.2.1.1	Transketolase	BLDA23_RS11000	LAZ97_RS09940
proB	2.7.2.11	Glutamate 5-Kinase	BLDA23_RS11660	LAZ97_RS10465
proC	1.5.1.2	Pyrroline-5-Carboxylate Reductase	BLDA23_RS11665	LAZ97_RS10470
ilvD	4.2.1.9	Dihydroxy-Acid Dehydratase	BLDA23_RS12585	LAZ97_RS11060
metA	2.3.1.31	Homoserine O-Succinyltransferase/O-Acetyltransferase	BLDA23_RS12610	LAZ97_RS11080
crr	2.7.1.-	Sugar Pts System Eiia Component	BLDA23_RS12760	LAZ97_RS11245
aspB	2.6.1.1	Aspartate Aminotransferase	BLDA23_RS12830	LAZ97_RS11315
dapB	1.17.1.8	4-Hydroxy-Tetrahydrodipicolinate Reductase	BLDA23_RS12895	LAZ97_RS11375
aroA	5.4.99.5	Chorismate Mutase	BLDA23_RS12900	LAZ97_RS11380
hisC	2.6.1.9	Histidinol-Phosphate Aminotransferase	BLDA23_RS12960	LAZ97_RS11445
trpA	4.2.1.20	Tryptophan Synthase Alpha Chain	BLDA23_RS12965	LAZ97_RS11450
trpB	4.2.1.20	Tryptophan Synthase Beta Chain	BLDA23_RS12970	LAZ97_RS11455
trpF	5.3.1.24	Phosphoribosylanthranilate Isomerase	BLDA23_RS12975	LAZ97_RS11460
trpC	4.1.1.48	Indole-3-Glycerol Phosphate Synthase	BLDA23_RS12980	LAZ97_RS11465
trpD	2.4.2.18	Anthranilate Phosphoribosyltransferase	BLDA23_RS12985	LAZ97_RS11470
trpE	4.1.3.27	Anthranilate Synthase Component	BLDA23_RS12990	LAZ97_RS11475
aroB	4.2.3.4	3-Dehydroquinate Synthase	BLDA23_RS13000	LAZ97_RS11485
aroC	4.2.3.5	Chorismate Synthase	BLDA23_RS13005	LAZ97_RS11490
serA	1.1.1.95	Phosphoglycerate Dehydrogenase	BLDA23_RS13190	LAZ97_RS11665
lysA	4.1.1.20	Diaminopimelate Decarboxylase	BLDA23_RS13370	LAZ97_RS11800
aroE	1.1.1.25	Shikimate Dehydrogenase	BLDA23_RS14720	LAZ97_RS13065

cysK	2.5.1.47	Cysteine Synthase	BLDA23_RS15295	LAZ97_RS13320
pheA	5.4.99.5	Chorismate Mutase	BLDA23_RS15610	LAZ97_RS13625
pheB	5.4.99.5	Chorismate Mutase	BLDA23_RS15615	LAZ97_RS13630
leuD	4.2.1.33	3-Isopropylmalate/(R)-2-Methylmalate Dehydratase Small Subunit	BLDA23_RS16050	LAZ97_RS14025
leuC	1.4.1.9	Leucine Dehydrogenase	BLDA23_RS16055	LAZ97_RS14030
leuB	1.1.1.85	3-Isopropylmalate Dehydrogenase	BLDA23_RS16060	LAZ97_RS14035
leuA	2.3.3.13	2-Isopropylmalate Synthase	BLDA23_RS16065	LAZ97_RS14040
ilvC	1.1.1.86	Ketol-Acid Reductoisomerase	BLDA23_RS16070	LAZ97_RS14045
ilvN	2.2.1.6	Acetolactate Synthase I/Iii Small Subunit	BLDA23_RS16075	LAZ97_RS14050
BCAT2	2.6.1.42	Branched-Chain Amino Acid Aminotransferase	BLDA23_RS16085	LAZ97_RS19625
lysC	2.7.2.4	Aspartate Kinase	BLDA23_RS16245	LAZ97_RS14170
gapA	1.2.1.13	Glyceraldehyde-3-Phosphate Dehydrogenase	BLDA23_RS16545	LAZ97_RS14430
pyk	2.7.1.40	Pyruvate Kinase	BLDA23_RS16615	LAZ97_RS14545
pfkA	2.7.1.11	6-Phosphofructokinase 1	BLDA23_RS16620	LAZ97_RS14550
argH	4.3.2.1	Argininosuccinate Lyase	BLDA23_RS16690	LAZ97_RS14615
argG	6.3.4.5	Argininosuccinate Synthase	BLDA23_RS16695	LAZ97_RS14620
pheA	5.4.99.5	Chorismate Mutase	BLDA23_RS16885	LAZ97_RS14875
cysK	2.5.1.47	Cysteine Synthase	BLDA23_RS16995	LAZ97_RS14985
dapE	3.5.1.18	Succinyl-Diaminopimelate Desuccinylase	BLDA23_RS17010	LAZ97_RS14990
asnB	6.3.5.4	Asparagine Synthase	BLDA23_RS17225	LAZ97_RS15220
pgi	5.3.1.9	Glucose-6-Phosphate Isomerase	BLDA23_RS18110	LAZ97_RS15720
dapF	5.1.1.7	Diaminopimelate Epimerase	BLDA23_RS18485	LAZ97_RS16160
thrB	2.7.1.39	Homoserine Kinase	BLDA23_RS18545	LAZ97_RS16195
thrC	4.2.3.1	Threonine Synthase	BLDA23_RS18550	LAZ97_RS16200

hom	1.1.1.3	Homoserine Dehydrogenase	BLDA23_RS18555	LAZ97_RS16205
eno	4.2.1.11	Enolase	BLDA23_RS19495	LAZ97_RS17395
gpmI	5.4.2.12	2,3-Bisphosphoglycerate-Independent Phosphoglycerate Mutase	BLDA23_RS19500	LAZ97_RS17400
pgk	2.7.2.3	Phosphoglycerate Kinase	BLDA23_RS19510	LAZ97_RS17410
gapA	1.2.1.13	Glyceraldehyde-3-Phosphate Dehydrogenase	BLDA23_RS19520	LAZ97_RS17415
cysK	2.5.1.47	Cysteine Synthase	BLDA23_RS19840	LAZ97_RS01095
hisIE	3.6.1.31	Phosphoribosyl-Atp Pyrophosphohydrolase	BLDA23_RS19845	LAZ97_RS17815
hisF	4.3.2.10	Imidazole Glycerol-Phosphate Synthase Subunit Hisf	BLDA23_RS19850	LAZ97_RS17820
hisA	5.3.1.16	Phosphoribosylformimino-5-Aminoimidazole Carboxamide Ribotide Isomerase	BLDA23_RS19855	LAZ97_RS17825
hisH	4.3.2.10	Midazole Glycerol-Phosphate Synthase Subunit Hish	BLDA23_RS19860	LAZ97_RS17830
hisB	3.1.3.15	Histidinol-Phosphatase	BLDA23_RS19865	LAZ97_RS17835
hisD	1.1.1.23	Histidinol Dehydrogenase	BLDA23_RS19870	LAZ97_RS17840
hisG	2.4.2.17	Atp Phosphoribosyltransferase	BLDA23_RS19875	LAZ97_RS17845
hisZ	-	Atp Phosphoribosyltransferase Regulatory Subunit	BLDA23_RS19880	LAZ97_RS17850
cysE	2.3.1.30	Serine O-Acetyltransferase	BLDA23_RS19895	LAZ97_RS17860
glyA	2.1.2.1	Glycine Hydroxymethyltransferase	BLDA23_RS20860	LAZ97_RS18810
fbaA	4.1.2.13	Fructose-Bisphosphate Aldolase, Class Ii	BLDA23_RS20985	LAZ97_RS18925
BCAT2	2.6.1.42	Branched-Chain Amino Acid Aminotransferase	BLDA23_RS21610	LAZ97_RS01410
ald	1.4.1.1	Alanine Dehydrogenase	BLDA23_RS22560	LAZ97_RS16010
